# From Venom to Vein: Factor VII Activation as a Major Pathophysiological Target for Procoagulant Australian Elapid Snake Venoms

**DOI:** 10.3390/toxins16100430

**Published:** 2024-10-06

**Authors:** Uthpala Chandrasekara, Abhinandan Chowdhury, Lorenzo Seneci, Christina N. Zdenek, Nathan Dunstan, Bryan G. Fry

**Affiliations:** 1 Adaptive Biotoxicology Lab, School of the Environment, University of Queensland, St. Lucia, QLD 4072, Australia; u.chandrasekara@uq.net.au (U.C.); abhinandan.choudhury@uq.edu.au (A.C.); l.seneci@student.uq.edu.au (L.S.); 2 School of the Environment, University of Queensland, St. Lucia, QLD 4072, Australia; christinazdenek@gmail.com; 3 Venom Supplies Pty Ltd., Stonewell Rd., Tanunda, SA 5352, Australia; nathan@venomsupplies.com

**Keywords:** venom, adaptation, coagulation, zymogen, molecular evolution, Factor Va, Factor VII, prothrombin, evolutionary biology

## Abstract

Australian elapid snake venoms are uniquely procoagulant, utilizing blood clotting enzyme Factor Xa (FXa) as a toxin, which evolved as a basal trait in this clade. The subsequent recruitment of Factor Va (FVa) as a toxin occurred in the last common ancestor of taipans (*Oxyuranus* species) and brown snakes (*Pseudonaja* species). Factor II (prothrombin) activation has been stated as the primary mechanism for the lethal coagulopathy, but this hypothesis has never been tested. The additional activation of Factor VII (FVII) by *Oxyuranus*/*Pseudonaja* venoms has historically been considered as a minor, unimportant novelty. This study aimed to investigate the significance of toxic FVII activation relative to prothrombin activation by testing a wide taxonomical range of Australian elapid species with procoagulant venoms. The activation of FVII or prothrombin, with and without the Factor Va as a cofactor, was assessed, along with the structural changes involved in these processes. All procoagulant species could activate FVII, establishing this as a basal trait. In contrast, only some lineages could activate prothrombin, indicating that this is a derived trait. For species able to activate both zymogens, Factor VII was consistently more strongly activated than prothrombin. FVa was revealed as an essential cofactor for FVII activation, a mechanism previously undocumented. Species lacking FVa in their venom utilized endogenous plasma FVa to exert this activity. The ability of the human FXa:FVa complex to activate FVII was also revealed as a new feedback loop in the endogenous clotting cascade. Toxin sequence analyses identified structural changes essential for the derived trait of prothrombin activation. This study presents a paradigm shift in understanding how elapid venoms activate coagulation factors, highlighting the critical role of FVII activation in the pathophysiological effects upon the coagulation cascade produced by Australian elapid snake venoms. It also documented the novel use of Factor Va as a cofactor for FVII activation for both venom and endogenous forms of FXa. These findings are crucial for developing better antivenoms and treatments for snakebite victims and have broader implications for drug design and the treatment of coagulation disorders. The research also advances the evolutionary biology knowledge of snake venoms.

## 1. Introduction

**Figure 1:**
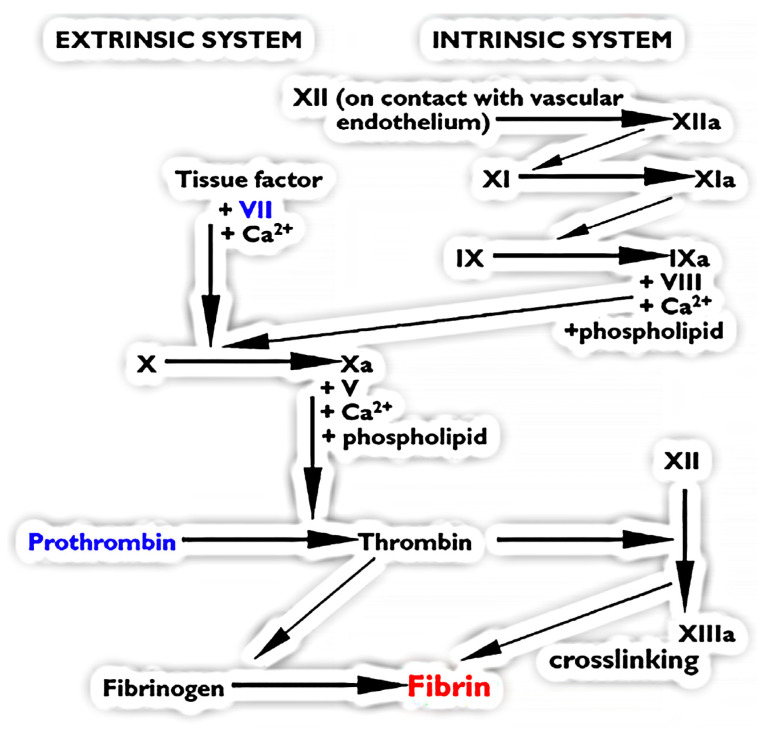
The clotting cascade showing the extrinsic and intrinsic pathways, which both ultimately trigger the formation of fibrin clots (**shown in red**). The two zymogens examined in this study (FVII and prothrombin) are **shown in blue**.

Vertebrate blood clotting is initiated by pathways consisting of successive steps of proteolytic activation of the zymogen forms of clotting enzymes into their active forms [1] (Figure 1). Zymogens, or proenzymes, are inactive enzymes that play a pivotal role in the coagulation cascade. They require activation to become functional enzymes that can catalyze the next step in the cascade. This activation typically occurs via a cleavage by another protease, which ensures that coagulation occurs rapidly and at the right place. The zymogen forms of clotting factors help to keep the system in check and prevent unwanted clot formation, which could lead to thrombosis, while conversely a lack of clot formation could lead to hemorrhage and hemorrhagic shock. The clotting cascade has three main pathways: extrinsic, intrinsic, and common. In the extrinsic pathway, trauma resulting in damage to vascular cells exposes Tissue Factor (TF), a glycoprotein on the surface of subendothelial cells, to Factor VII (FVII) present in plasma, with TF converting FVII into the activated form Factor VIIa (FVIIa). The TF:FVIIa complex subsequently converts Factor X (FX) into its activated form Factor Xa (FXa). In the intrinsic pathway, Factor XII (FXII) is converted into its active form Factor XIIa (FXIIa) in response to the detection of endothelial surface abnormalities or recognition of foreign material (especially lipids such as bacterial lipopolysaccharides and other negatively charged molecules such as polyphosphates released by activated platelets). FXIIa in turn mediates the conversion of Factor XI to its activated form Factor XIa (FXIa), which in turn converts Factor X into its activated form Factor Xa (FXa). The common pathway is defined as all of the steps downstream from the activation of FX into FXa. FXa in the presence of cofactors calcium and phospholipid forms a complex with the activated form of Factor V (FVa). The FXa:FVa complex, also known as the prothrombinase complex, converts Factor II (FII, but more commonly referred to as prothrombin, with this terminology used for the remainder of this article to avoid confusion) into its active form Factor IIa (FIIa, but more commonly referred to as thrombin, with this terminology used for the remainder of this article to avoid confusion). Thrombin in turn converts Factor I (FI, but more commonly referred to as fibrinogen, with this terminology used for the remainder of this article to avoid confusion) into clot-forming fibrin. Fibrinogen is a soluble protein made up of three chains (Aα, Bβ, and γ), while the resulting cross-linked fibrin is insoluble. The insolubility of fibrin is key to its ability to form the strands that make up the core of the formed clot.

Given the delicate balance of the hemostatic system and its intimate relationship to both wound repair and immune responses, it is not surprising that venomous organisms have evolved mechanisms to manipulate it for their advantage. In snakes, procoagulant toxicity (venom-induced activation of clotting factors) has been documented in a wide variety of species, convergently evolving in multiple families, but all leading to consumptive coagulopathy [2,3,4,5,6,7,8,9]. The procoagulant toxicity of *Atractaspis* species within the Lamprophiidae family is due to the activation of FX [10]. Both FX and prothrombin are activated by metalloproteases in viperid snakes [11,12]. *Porthidium volcanicum* is unique amongst viperid snakes in being procoagulant through the activation of FVII [13]. In addition to activating coagulation enzyme FX, some viperid procoagulant snake venoms also activate FV through the use of kallikrein-type serine proteases [14,15,16,17,18,19]. *Rhabdophis subminiatus* within the natricinae subfamily of the Colubridae family has a venom that uniquely activates multiple factors including prothrombin, FVII, FIX, FX, FXI, and FXII, with FVII activated at a much higher level than any of the other factors [20].

In Australian elapid snakes, the procoagulant function results from the unique recruitment of the endogenous clotting factors FXa and FVa as weaponized forms for use as toxins. Post-envenomation, the venom FXa homologue couples with the bite victim’s endogenous FVa to form a prothrombinase complex (FXa:FVa), mediated by the cofactors calcium and phospholipid [21,22,23,24,25,26,27]. This complex then converts prothrombin into active thrombin, which rapidly converts fibrinogen into fibrin clots [25]. While FXa was recruited at the base of Australian elapid clade, it maintained low levels prior to multiple, convergent amplifications on at least seven occasions: *Cryptophis* genus, *Demansia* genus, *Hemiaspis* genus, *HoplocephalusNotechis*/*Paroplocephalus*/*Tropidechis* clade, *Oxyuranus*/*Pseudonaja* clade, *Pseudechis porphyriacus* uniquely within *Pseudechis* genus, and *Suta* genus [24,28,29,30]. As these other lineages have independently amplified the ancestral FXa toxin trait and thus have differential molecular evolutionary patterns, it is unlikely they have all taken the same constrained functional trajectory. Consistent with this, significant variation in clotting ability and cofactor interactions have been documented across these lineages [21,24,27]. Although the role of FXa as a prothrombin activator in these venoms is well recognized [31,32,33,34,35,36,37], the full range of actions by venom-modified FXa remains poorly understood and under-researched. Prothrombin activation has only been tested for species within the genera *Hoplocephalus*, *Notechis*, *Oxyuranus*, *Pseudonaja*, and *Tropidechis* [31,36,38,39,40,41,42,43,44,45,46].

The Australian elapid genera *Oxyuranus* (taipans) and *Pseudonaja* (brown snakes) are unique in that in addition to the FXa found in other Australian snake venoms, their common ancestor also recruited the activated form of FVa into their venoms [47,48]. This removed the reliance on endogenous FVa to form the FXa:FVa prothrombinase complex [22]. The venom form of this complex has acquired several unique gain-of-function features that make it several orders of magnitude more potent than the endogenous prothrombinase complex [49]. This innovation contributes to the very high toxicity of these genera when compared to other venomous snakes [50]. The mechanisms by which the venom form of FVa escapes hemostatic control [51] is what facilitates the devastating pathology of envenomation by these genera. Previous studies revealed that the purified FXa:FVa prothrombin activator from *Oxyuranus scutellatus venom* was able to activate FVII [52], with a latter study documenting that this was a trait shared with *Pseudonaja* venoms and, therefore, was present in the last common ancestor of the *Oxyuranus*/*Pseudonaja* clade [21]. However, the ability of other Australian procoagulant snake venoms (which contain only FXa in their venom) to activate FVII has not been investigated.

This study sought to understand the procoagulant toxicity of Australian elapid snakes by testing a wide variety of venoms, including those with only FXa in the venom and also FXa:FVa-containing venoms, for their relative ability to activate prothrombin and Factor VII. Understanding the specific mechanisms by which elapid venoms activate coagulation factors such as prothrombin and FVII not only helps understand the selection pressures shaping venom evolution but also is crucial for the development of more effective antivenoms and treatments for snakebite victims, such as testing whether variable factor activation is responsible for the differential response of Australian procoagulant snakes to Tiger Snake antivenom [29]. Moreover, these insights have broader implications for coagulation biology, potentially informing new approaches to managing coagulation disorders in humans, through the utilization of novel venom enzymes as starting substrates in the drug design and development pipeline.

## 2. Results and Discussion

**Figure 2:**
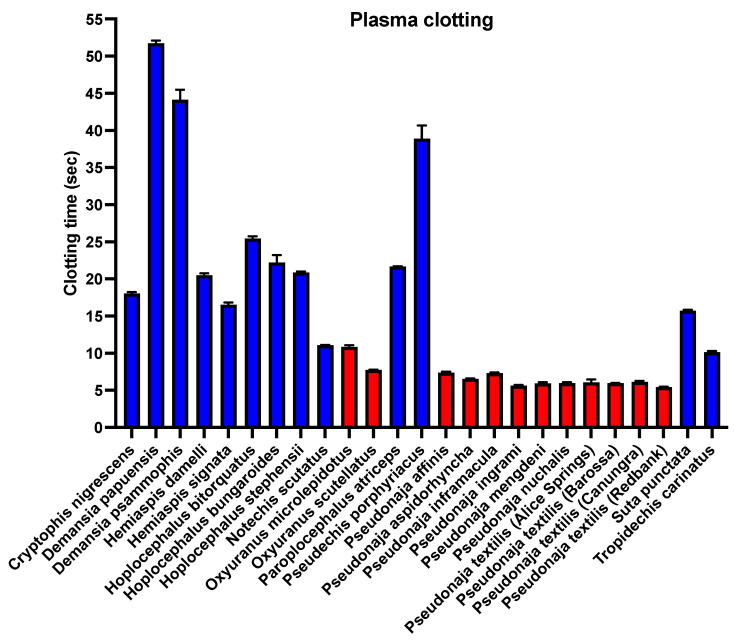
Plasma clotting effects of venoms containing only weaponized FXa (blue) and those containing both weaponized FXa and weaponized FVa (red). Lower values indicate faster clotting and thus greater potency. Values are N = 4 calculated as mean and standard deviation. Spontaneous clotting control time was 431.5 ± 7.7 s. Graphed values are in Supplementary File S1.

Consistent with Factor Xa being recruited at the base of the Australian radiation [25,53,54], the ability to clot plasma was shown to be a basal trait [29], with all venoms significantly accelerating clotting (Figure 2) relative to the spontaneous clotting control time of 431.5 ± 7.7 s.

**Figure 3:**
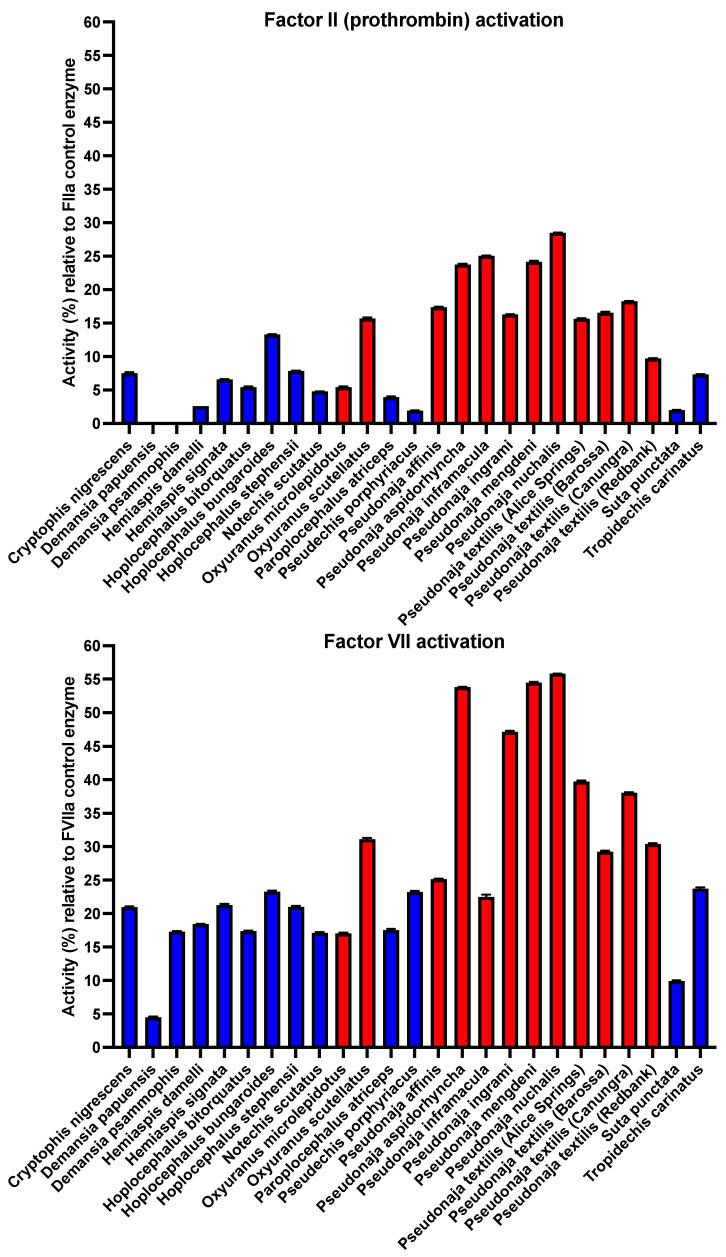
Zymogen activation effects of venoms containing only weaponized FXa (blue) and those containing both weaponized FXa and weaponized FVa (red). Higher values indicate greater potency. Note: for allow a comparison across venoms, both graphs are scaled relative to the point of the greatest impact across the graphs. Values are area under the curve, N = 4, calculated as mean and standard deviation. *Y*-axis values are % relative to the positive control (FVIIa or thrombin), which are set to 100%. Graphed values are in Supplementary File S1.

**Figure 4:**
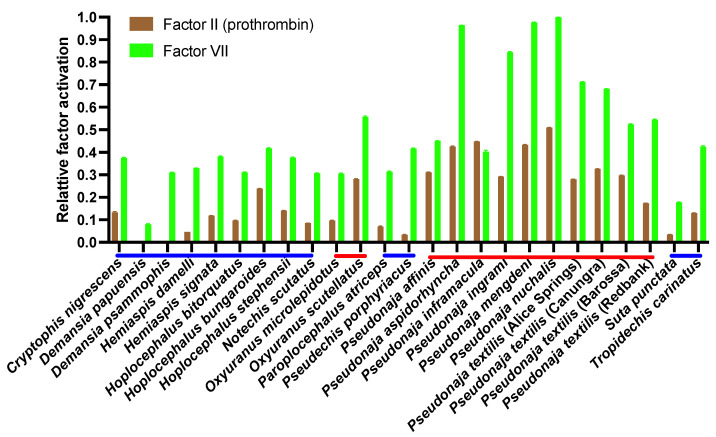
Relative ability to activate the prothrombin (brown) and Factor VII (green) zymogen activation effects of venoms containing only weaponized FXa (blue lines above taxa names) and those containing both weaponized FXa and weaponized FVa (red lines above taxa names). Higher values indicate greater potency. Relative differences between pairs for each species were statistically significant (*p* < 0.001). *Y*-axis values are Figure 3 values normalized relative to the most potent venom (*Pseudonaja nuchalis*). Graphed values are in Supplementary File S1.

**Figure 5:**
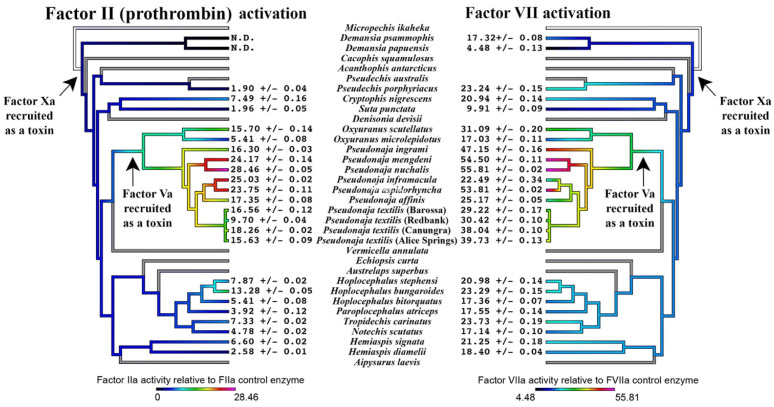
Ancestral reconstruction of prothrombin and Factor VII zymogen activation. Organismal phylogeny based on Lee [63]. Toxin recruitment phylogenetic positions are shown. Values (% relative to control enzyme) are area under the curve, N = 4, calculated as mean and standard deviation. Branch color scheme has a colder color for weaker activity and a warmer color for stronger activity. Non-procoagulant lineages within the Australian snake radiation are shown with gray branches in order to place convergent amplification of the procoagulant trait into the full evolutionary context. N.D. = not detectable.

However, subsequent tests for the relative activation of prothrombin and FVII revealed significant differences in the relative ability to activate FVII versus prothrombin, with all venoms being able to activate FVII to some degree, but not all being able to activate prothrombin (Figure 3). A pattern emerged that FVII was more strongly activated than prothrombin (Figure 4). Phylogenetic mapping revealed that FVII activation is a basal trait, but that prothrombin activation is a derived trait present only in some lineages (Figure 5). Consistent with a previous study, the FXa:FVa-containing *Oxyuranus* and *Pseudonaja* venoms were able to activate both human FVII and prothrombin [21,31,41,52]. In contrast, none of species that contained only FXa in their venoms (lacking FVa) were able to activate prothrombin in the absence of the cofactor Factor Va, consistent with previous documentation of FVa being an essential cofactor for FXa–venom activity [25,40,47,55,56,57]. Notably, these same FXa-only venoms also required the addition of human FVa to activate FVII (Figure 3). This is the first documentation that FVa is an essential cofactor for the activation of FVII.

Consistent with FVII activation being the ancestral trait, the species that were able to activate both FVII and prothrombin were significantly stronger activators of FVII than the derived trait of prothrombin activation (Figure 4 and Figure 5). The only exception was *Pseudonaja inframacula*, which was a marginally stronger activator of prothrombin (but still a potent activator of both zymogens). Congruent with plasma clotting results [29] (Figure 2), the venoms that contain Factor Va (*Oxyuranus* and *Pseudonaja* species) were more potent in activating either FVII or prothrombin than the venoms that contain only FXa (Figure 5).

As FVII is a basal trait, we tested the ability of the endogenous human FXa:FVa complex to activate FVII. This revealed that this complex is indeed able to activate FVII, having a potency of 6.28 +/− 0.042% relative to the positive control enzyme. This places it in the range of the lowest activity of snake venoms (4.48 +/− 0.13 for *Demansia papuensis* and 9.91 +/− 0.09 *Suta punctata*). It would be expected that the relative potency of human FXa:FVa to activate FVII would be lower than these values since human FXa:FVa was tested at the same mass as the snake venoms. However, it has been shown that the FXa concentration in Australian snake venoms is not high despite the procoagulant potency, with it being as low as 3% in *Hoplocephalus stephensi* venom, 5% in *Notechis scutatus* venom, and 5% for *Tropidechis carinatus* venom, but with the venom forms being much more active in activating prothrombin than endogenous FXa [47,58]. Regardless of the relative potency between endogenous FXa and venom FXa in activating FVII, as FX is activated by FVIIa in the clotting cascade [1], the ability of endogenous FXa to in turn activate FVIIa is revealed in this study as a human clotting cascade feedback loop that has not been previously documented.

**Figure 6:**
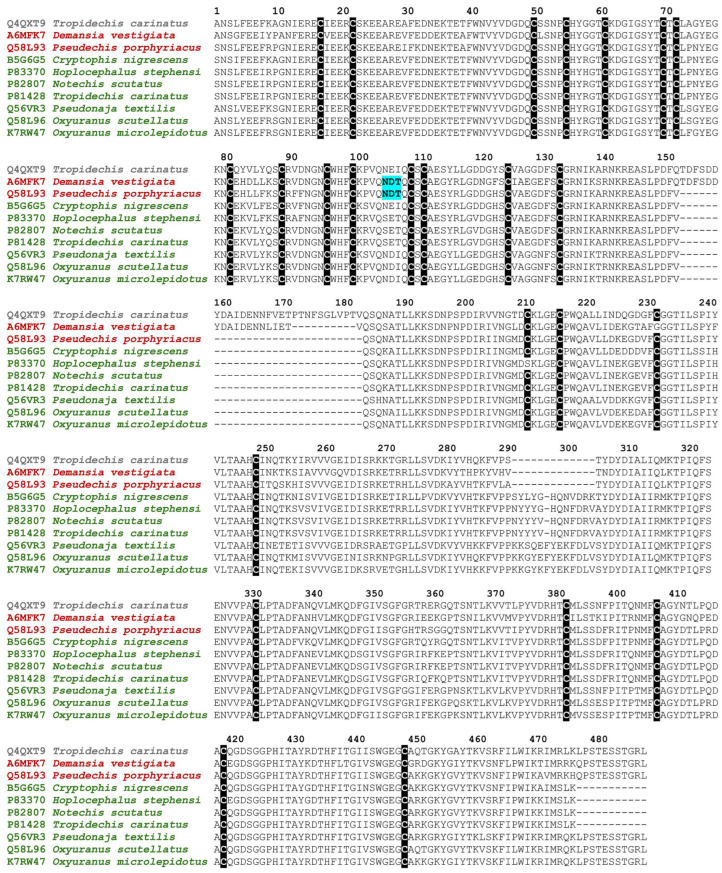
Sequence alignment of FXa enzymes. **Gray** is a liver produced Factor X used in circulating plasma. **Brown** are the venom forms: unable to activate prothrombin in brown with *Demansia vestigiata* acting as a proxy for the *D. papuensis* and *D. psammophis* venoms examined in this study as sequences are currently not available for these species or only weakly active (*Pseudechis porphyriacus*). **Green** are the venom forms able to strongly activate prothrombin (*Cryptophis*, *Hoplocephalus*, *Notechis*, *Tropidechis*, *Pseudonaja*, and *Oxyuranus*). Cysteines are highlighted in black. The NX (S or T) glycosylation tri-amino acid motif shown in blue highlight (where X = any amino acid except for cysteine or proline). Note: signal peptide sequences removed to save space.

Comparing bioactivity patterns relative to venom FXa sequence motifs (Figure 6) revealed a structure–activity congruence. While species within the most basally diverging procoagulant genus (*Demansia*) were unable to activate prothrombin, the next diverging procoagulant lineage (*Pseudechis porphyriacus*) was only able to weakly activate this zymogen. This is congruent with both species posing a glycosylation motif at positions 105–107 that is not found in the other species. N-glycosylation is conferred by changes in the amino acid sequence: the tripeptide motif NX (S/T) emerges so that asparagine is followed by any amino acid (except cysteine (C) or proline (P)) which is, in turn, followed by either serine or threonine [59,60,61,62]. Glycosylation at other sites has been shown to be an important functional feature for FXa enzymes present in Australian elapid snake venoms [32,33]. As such, it is a testable hypothesis for future research using recombinant forms that the glycosylation at this novel site is responsive for the lack of meaningful activity by *Demansia* and *Pseudechis* FXa enzymes on prothrombin. Congruence with *P. porphyriacus* having a slight activity was not found for *Demansia* venoms, and a sequence deletion [29] at positions 153–184 (Figure 6) occurred after *Demansia* split off from the stem group, but before the divergence by *Pseudechis* occurred. An important caveat is that this hypothesis must be experimentally validated in future studies using recombinantly expressed sequence variations to ascertain the structure–activity relationships. Congruent with the increased prothrombin activation potency subsequent to the divergence by both *Demansia* and *Pseudechis*, an insertion at positions 202–305 [29] is found in these more derived species (Figure 6) that share a common ancestor. Again, this hypothesis must be experimentally validated in future studies using recombinantly expressed sequence variations to ascertain the structure–activity relationships.

These results document a previously unknown contribution to the procoagulant potency of Australian elapids. Previously, prothrombin activation was considered as the primary mechanism and Factor VII regarded as an unimportant novelty. This is reflected by the abundance of research into prothrombin activation [31,32,33,34,35,36,37], and only two studies into Factor VII activation that were 25 years apart (one in 1992, the other in 2017) [21,52]. Consequently, the relative contribution by activation of each factor has never been tested. The results in this study are the first such comparison and documentation that Factor VII is activated more strongly than prothrombin. It is also the first documentation of the requirement of FVa as a cofactor for FVII activation. While prothrombin levels (1.4 μM) in the plasma are in vast excess (~140-fold) over FVII levels (10 nM), this is offset by FVII being two steps above prothrombin in the clotting cascade. As Factor VIIa activates Factor X zymogen, FXa in turn activates prothrombin zymogen. Each Factor VIIa is able to activate multiple FX zymogens, and each FXa is able to activate multiple prothrombin zymogens. Therefore, the venom-induced activation of a single FVII zymogen would result in the generation of more endogenous thrombins than by the activation of a single prothrombin zymogen, leading to the generation of more fibrin clots. This therefore negates the relative stoichiometric difference between FVII and prothrombin circulating in the plasma, making FVII a viable pathophysiological target.

This unique venom specialization in Australian elapids is a result of evolutionary pressures that have shaped these venoms to suit the specific dietary and environmental requirements of these snakes. The procoagulant properties inherent to Australian elapid venoms have been notably enhanced through convergent evolution, indicating multiple independent evolutionary events where similar traits have been amplified. This suggests strong selective pressures favoring venoms that can more efficiently disrupt the hemostasis of prey, which leads to exceptionally rapid immobilization and death. The recruitment of Factor Xa (FXa) into the venom composition marked a significant evolutionary development among Australian elapids. However, the effectiveness of venom-derived FXa is inherently limited by its dependency on binding with Factor Va (FVa) in a 1:1 ratio, whether for activation of prothrombin or FVII. This requirement posed a significant limitation on the venom’s coagulation-inducing efficiency due to the requirement of an intermediate binding step. The evolutionary introduction of FVa directly into the venom of the *Oxyuranus*/*Pseudonaja* last common ancestor represents a profound adaptive response to the this bottleneck. By bypassing the need for FXa to find and bind FVa within the victim’s bloodstream, these snakes enhanced their venom’s capability to trigger rapid and uncontrollable coagulation, thus improving their effectiveness in subduing prey.

Understanding the specific mechanisms by which elapid venoms activate coagulation factors such as FVII and prothrombin is crucial for the development of more effective antivenoms and treatments for snakebite victims. Such variations in clotting factor activation may underpin the antivenom variability noted previously [29]. Moreover, these insights have broader implications for coagulation biology, potentially informing new approaches to managing coagulation disorders in humans, as these enzymes may be of use in diagnostic kit development or as lead compounds in drug design and development.

## 3. Materials and Methods

### 3.1. Venom Stocks

Venom work was undertaken with University of Queensland Biosafety Approval (#IBC134BSBS2015) and University of Queensland Animal Ethics Approval (15 March 2021/AE000075). Venoms of adult snakes (pools of N = 4, mixed sex) were supplied by licensed biotechnology company Venom Supplies Pty Ltd., Tanunda, South Australia. Species studied were *Cryptophis nigrescens* (Bunya, QLD, Australia), *Demansia olivacea* (Brisbane, QLD, Australia), *Demansia papuensis* (Cairns, QLD, Australia), *Demansia psammophis* (Brisbane, QLD, Australia), *Demansia vestigiata* (Cairns, QLD, Australia), *Hemiaspis damelii* (Glen Morgan, QLD, Australia), *Hemiaspis signata* (Brisbane, QLD, Australia), *Hoplocephalus bitorquatus* (Texas, QLD, Australia), *Hoplocephalus bungaroides* (Sydney, NSW, Australia), *Hoplocephalus stephensi* (Brisbane, QLD, Australia), *Notechis scutatus* (Mt Gambier, SA, Australia), *Oxyuranus microlepidotus* (Boulia, QLD, Australia), *Oxyuranus scutellatus* (Cooktown, QLD, Australia), *Paroplocephalus atriceps* (Lake Cronin, WA, Australia), *Pseudechis porphyriacus* (Brisbane, QLD, Australia), *Pseudonaja affinis* (Perth, WA, Australia), *Pseudonaja aspidorhyncha* (Dubbo, NSW, Australia), *Pseudonaja inframacula* (Yorke Pen SA), *Pseudonaja ingrami* (Barkly Tableland, QLD, Australia), *Pseudonaja mengdeni* (Alice Springs, NT, Australia), *Pseudonaja nuchalis* (Darwin NT, Australia), *Pseudonaja textilis* (Alice Springs, NT, Australia), *Pseudonaja textilis* (Barossa, SA, Australia), *Pseudonaja textilis* (Canungra, QLD, Australia), *Pseudonaja textilis* (RedBank, QLD, Australia), *Suta punctata* (Kununurra, WA, Australia), and *Tropidechis carinatus* (Brisbane, QLD, Australia).

### 3.2. Clotting Time

Plasma studies were undertaken with University of Queensland Biosafety Approval (#IBC134BSBS2015), University of Queensland Human Ethics Approval (2024/HE000675), and Australian Red Cross Research Agreement (# 22-05QLD-06). Fresh plasma pools from multiple donors of both sexes were aliquoted into 1 mL volumes, flash frozen in liquid nitrogen, and stored at −80 °C until needed. Coagulation tests were undertaken on a STA-R Max^®^ analyser (Stago, Asnières sur Seine, France) as previously described [29]. A 0.1 mg/mL venom stock (diluted with Owren-Koller (OK) buffer (Stago Cat# 00360)) was diluted to run at the reaction condition of 20 μg/mL. Later, 50 μL of venom, 50 μL of 25 mM CaCl_2_ (Stago Cat# 00367), 50 μL of phospholipid (Stago Cat# 00597, dissolved in OK buffer), and 25 μL of OK buffer were incubated together for 120 s. Then, 75 μL of plasma was added to bring the total reaction volume to 250 μL, and clotting was measured by the machine. The negative control (spontaneous control) was substituted for venom with 1:1 deionised water:Glycerol in OK buffer. The positive control was incubated 50 μL of Kaolin (Stago Cat# 00597), 50 μL of phospholipid (Stago Cat# 00597), 25 μL of OK buffer (Stago Cat# 00360), and 75 μL of plasma (Australian Red Cross) for 120 s and then 50 μL CaCl_2_ (25 mM) was added, and clotting was measured. The machine tested 8 concentrations of venom to generate a venom dilution curve. Positive and negative controls were performed in triplicate at 20 μg/mL concentration to ensure that optimal plasma and reagents were used.

### 3.3. Zymogen Activation

Prothrombin and FVII zymogen activation studies were undertaken in 384-well plates (black, lot#1171125, Nunc™ Thermo Scientific, Rochester, NY, USA) using Fluoroskan Ascent™ (Thermo Scientific, Vantaa, Finland), based on the reaction stoichiometry in the “Blood Clotting Factor Activation Assay” zymogen activation protocol of Seneci [64]. Plate setups contained blanks, zymogen controls, activated factor control, venom-only controls, and venom + zymogen experiments, all containing an enzyme buffer without calcium (150 mM NaCl and 50 mM Tri-HCl (pH 7.3)). The reaction composition conditions varied between FVII and prothrombin tests due to the extremely high affinity thrombin had for the fluorometric substrate, so stoichiometric conditions were scaled uniformly. Specific protocol details are as follows:

#### 3.3.1. Factor VII Activation Setup

##### Blank Control Wells

- 10 μL phospholipid (Stago 00597)
- 20 μL of enzyme buffer without calcium (150 mM NaCl, and 50 mM Tri-HCl (pH 7.3)

##### Zymogen Control Wells

- 10 μL phospholipid (Stago 00597)
- 10 μL of 10 μg/mL FVII (Prolytix #HCVII-0030)
- 10 μL of enzyme buffer without calcium (150 mM NaCl, and 50 mM Tri-HCl (pH 7.3)

##### Activated Enzyme Control Wells

- 10 μL phospholipid (Stago 00597)
- 10 μL of 10 μg/mL FVIIa (Prolytix #HCVII-0030)
- 10 μL of enzyme buffer without calcium (150 mM NaCl, and 50 mM Tri-HCl (pH 7.3)

##### Venom Control Wells

- 10 μL phospholipid (Stago 00597)
- 10 μL of 1 μg/mL venom
- 10 μL of enzyme buffer without calcium (150 mM NaCl, and 50 mM Tri-HCl (pH 7.3)

##### Experimental Wells

- 10 μL phospholipid (Stago 00597)
- 10 μL of 10 μg/mL FVII (Prolytix #HCVII-0030)
- 10 μL of venom as separate experiments to test the role of FVa:
  ○ 10 μL of 1 μg/mL venom **or**
  ○ 10 μL consisting of:
    ▪ 1 μg/mL venom **+**
    ▪ 4 µg/mL Factor Va (Prolytix #HCVA-0110)

#### 3.3.2. Prothombin Activation Reaction Setup

##### Blank Control Wells

- 10 μL phospholipid (Stago 00597)
- 20 μL of enzyme buffer without calcium (150 mM NaCl, and 50 mM Tri-HCl (pH 7.3)

##### Zymogen Control Wells

- 10 μL phospholipid (Stago 00597)
- 10 μL of 1 μg/mL prothrombin (Prolytix HCP-0010)
- 10 μL of enzyme buffer without calcium (150 mM NaCl, and 50 mM Tri-HCl (pH 7.3)

##### Activated Enzyme Control Wells

- 10 μL phospholipid (Stago 00597)
- 10 μL of 1 μg/mL thrombin (Prolytix HCT-0020)
- 10 μL of enzyme buffer without calcium (150 mM NaCl, and 50 mM Tri-HCl (pH 7.3)

##### Venom Control Wells

- 10 μL phospholipid (Stago 00597)
- 10 μL of 0.1 μg/mL venom
- 10 μL of enzyme buffer without calcium (150 mM NaCl, and 50 mM Tri-HCl (pH 7.3)

##### Experimental Wells

- 10 μL phospholipid (Stago 00597)
- 10 μL of 1 μg/mL prothrombin (Prolytix HCP-0010)
- 10 μL of venom as separate experiments to test the role of FVa:
  ○ 10 μL of 0.1 μg/mL venom **or**
  ○ 10 μL consisting of:
    ▪ 0.1 μg/mL venom **+**
    ▪ 0.4 µg/mL Factor Va (Prolytix #HCVA-0110)

#### 3.3.3. Faction Activation Reaction Commencement

Subsequently, reactions were started through automatic pipetting into each well of 70 μL of buffer (5 mM CaCl_2_,150 mM NaCl, and 50 mM Tri-HCl (pH 7.3)). The calcium in the buffer is an essential cofactor for FVII and prothrombin activation, thereby acting as a trigger. The buffer included the fluorescent substrate ES011 (Boc-Val-Pro-Arg-AMC; Boc = t-Butyloxycarbonyl; AMC = 7-Amino-4-methylcoumarin; R & D systems, catalogue# ES011, Minneapolis, Minnesota) added at a buffer/substrate volumetric ratio of 500:1. Excitation wavelength was 390 nm, and emission wavelength was 460 nm. Plates were run at 37 °C. Resulting values obtained for blank conditions were subtracted from reactions from the same plate. In addition, to control for any venoms acting directly on the substrate, thus artificially increasing the fluorescence values, venom-only values were subtracted from data obtained from wells containing the same venom incubated with zymogens.

### 3.4. Ancestral State Reconstruction

The phylogenetic tree used was based on a previously published species tree [63] and manually recreated using the Mesquite software (version 3.2) and then imported to Rstudio using the APE package [65]. Ancestral states were estimated for all traits using maximum likelihood as implemented in the contMap function of the R package [66] using the following command lines:

library(maps)

library(phytools)

data<-read.csv(file.choose())

dat<-data

mapvar<-dat$var

names(mapvar)<-dat$species

tree<-read.tree(file.choose())

asr<-contMap(tree,mapvar,plot = F)

plot(setMap(asr,col = c(1,4,5,3,7,2,6)),lwd = 3)

### 3.5. FXa Molecular History Reconstruction

Sequences were aligned in Aliview, and phylogenetic reconstruction was undertaken in MrBayes using the following nexus block:

log start replace;

set autoclose = no nowarn=no;

lset applyto = (all) nst=6 rates = invgamma;

prset applyto = (all) aamodelpr = mixed;

unlink revmat = (all) shape = (all) pinvar = (all)statefreq = (all) tratio = (all);

showmodel;

mcmc ngen = 15000000 printfreq = 1000 samplefreq=100 nchains = 4 temp = 0.2 checkfreq = 50000 diagnfreq = 1000 stopval = 0.01 stoprule = yes;

sumt relburnin = yes burninfrac = 0.25 contype = halfcompat;

sump relburnin = yes burninfrac = 0.25;

outgroup 1;

## Data Availability

All data are contained within the figures.

## Supplementary Materials

The following supporting information can be downloaded at: https://www.mdpi.com/article/10.3390/toxins16100430/s1, Supplementary File S1 contains the graphed values for Figure 2, Figure 3 and Figure 4.
